# Laser Confocal Microscopy May Be a Useful Tool in Neuropathological Intraoperative Examination

**DOI:** 10.3390/diagnostics15222936

**Published:** 2025-11-20

**Authors:** Deborah Dardano, Anna Bilotta, Gianmarco Gallucci, Carlo Gentile, Giuseppe Riganati, Antonio Veraldi, Domenico Policicchio, Maria Teresa Nevolo, Alberto V. Filardo, Anna Maria Lavecchia, Giuseppe Donato

**Affiliations:** 1 Department of Health Sciences, University “Magna Græcia” of Catanzaro, 88100 Catanzaro, Italy; 2 “R. Dulbecco” University Hospital, 88100 Catanzaro, Italy; 3 Anatomical Pathology Unit, University Hospital “R. Dulbecco”, 88100 Catanzaro, Italy

**Keywords:** Histolog^®^ Scanner, laser confocal microscopy, intraoperative pathological examination, brain tumors surgery, margins

## Abstract

**Background and Clinical Significance:** The paper investigates the use of the Histolog^®^ Scanner, a confocal microscopy–based device, as a potential tool for intraoperative neuropathological diagnosis of brain tumors. Traditional intraoperative diagnosis, relying on frozen sections and squash preparations, can introduce artifacts and consume valuable tissue. The Histolog^®^ Scanner offers a plug-and-play solution capable of acquiring high-resolution images of fresh tissue surfaces in minutes while preserving tissue for further histological or molecular analyses. **Cases Presentation:** Three clinical cases—two women and one-man, mean age 57.3 years—undergoing neurosurgery for distinct brain lesions were included. Tissue samples were immersed in fluorescent dye, rinsed, and immediately analyzed with the Histolog^®^ Scanner before standard intraoperative histopathology. In the first case, a glioblastoma wild-type, traditional methods struggled to define tumor margins, whereas the device provided rapid, detailed imaging to guide resection. In the second case, a meningioma, the scanner confirmed lesion identity quickly, eliminating the need for a cryostat and reducing artifacts. In the third case, a brain metastasis, integration with cytological apposition allowed simultaneous assessment of lesion margins and nature without freezing the tissue. **Conclusions:** The Histolog^®^ Scanner demonstrated multiple advantages: rapid intraoperative use, clear margin visualization, preservation of tissue for subsequent analyses, reduce unnecessary resection, thereby helping to lower the risk of recurrence. This device may complement standard intraoperative methods, enhancing diagnostic accuracy and influencing postoperative treatment planning. Overall, the Histolog^®^ Scanner represents an innovative tool combining speed, precision, and tissue preservation, suggesting a promising role in establishing a new standard for intraoperative neurosurgical diagnosis.

## 1. Introduction

Intraoperative pathological examination in neurosurgery is a compelling challenge due to the sharp observation and reasoning it requires. First and foremost, the patient’s clinical data and neuroradiological imaging must be fully available because they are an integral part of the procedure itself and greatly facilitate it. The intraoperative diagnosis can be more or less precise and consistent with the definitive one, depending on the type of lesion studied and the techniques used [1]. Classic techniques—frozen sections, squashing, and touch preparations—must be available and familiar to the pathologist, but they are not always used in the same procedure. Procedural decisions also depend on sampling methods, which yield different amounts of material. Stereotactic biopsy and certain modern neuronavigation techniques can provide small amounts of material for examination, but also to preserve for definitive examination after paraffin embedding, comprising eventual molecular analysis. Moreover, in the era of targeted therapies, achieving a safe maximum resection remains a critical prognostic factor in diffuse gliomas and represents the goal for neurosurgeons managing these tumors. Fluorescence-guided surgery, using 5-aminolevulinic acid or even sodium fluorescein, significantly increases the extent of resection of high-grade gliomas without causing further harm to the patient. Supramaximal resection beyond the contrast-enhancing tumor margins represents an emerging surgical strategy for patients with newly diagnosed glioblastoma wild-type. This is especially indicated in elderly patients, when the lesion is not in eloquent areas [2,3,4]. From a practical standpoint, integrating a rapid intraoperative assessment with these new surgical requirements seems difficult; however, intraoperative examinations conducted with laser confocal microscopy techniques have recently been introduced in surgical pathology for similar purposes. The Histolog^®^ Scanner (SamanTree Medical SA, Lausanne, Switzerland) is a plug-and-play device that can be installed and used in the operating room or pathology laboratory. It is a confocal microscopy device designed for imaging the surface of excised human tissue samples to visualize morphological microstructures. Scanning samples ranging from a few millimeters to approximately 17 cm^2^ takes less than a minute. Easy-to-read images of the entire surface are quickly available for studying the tumor and its resection margins. To date, the main applications of this instrument have been in breast and prostate surgery, and in very few other fields [5,6,7,8,9,10,11,12,13,14,15,16,17,18]. The examination by Histolog^®^ Scanner is very interesting because of its speed and because it preserves the characteristics of the sample, which can be used for all normal histopathological diagnostic procedures. Cases of brain lesions we examined suggest that these procedures could already lead to a significant leap in quality in this challenging diagnostic area. Such confocal microscopy techniques can be helpful in neuropathology for the diagnosis of resection margins of tumor lesions and, surprisingly, also in the diagnosis of the nature of the lesions, also allowing material otherwise used for possible frozen sections to be saved. Equipment such as the Histolog^®^ Scanner appears to be able to provide real-time information regarding intraoperative resection margins, even in neuropathology, for lesions such as gliomas, metastases, or meningiomas, just as has been the case to date for breast or prostate surgery. So, we conducted a pilot study to test the feasibility of a larger research design and to identify potential problems before conducting a full study.

## 2. Materials and Methods

To test the Histolog^®^ Scanner’s ability to support the intraoperative diagnosis of brain tumors and assess resection margins, we selected three patients undergoing brain tumor surgery. The expected diagnostic findings for these patients, both clinically and radiologically, were a high-grade glial tumor, a metastatic brain tumor, and an intracranial meningioma. The purpose of this selection was to evaluate the feasibility of using the device in neuropathological diagnostics, leaving the exploration of larger case series for a possible subsequent study.

Two patients were female and one male, with a mean age of 57.3 years (range 45–75 years). Medical and clinical histories were collected. Neurological examinations were performed before and after surgery.

**Figure 1:**
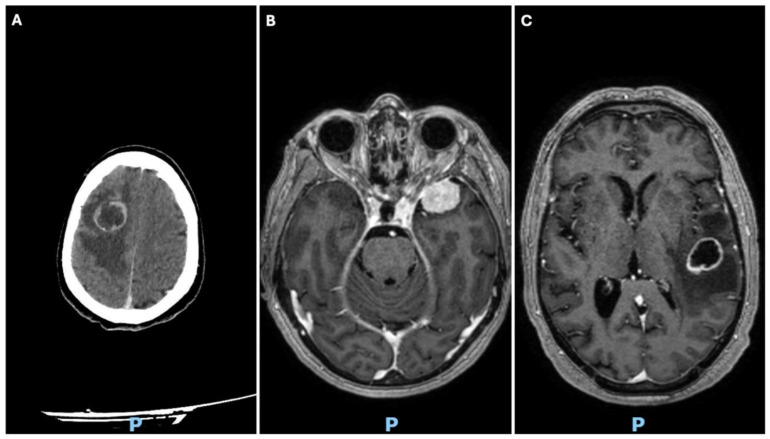
(**A**). Brain CT scan showing right frontoparietal necrotic lesion with ring-shaped contrast enhancement and perilesional edema. (**B**). T1-weighted MRI images showing the presence of a lesion with uneven intensity in the left sphenoid wing region. (**C**). T1-weighted MRI of the brain showing a left temporo-parietal lesion with ring-shaped hyperintensity and perilesional edema.

All patients underwent conventional radiography (X-ray), magnetic resonance imaging (MRI), and computed tomography (CT) preoperatively to make a provisional diagnosis [Figure 1]. All patients underwent surgical treatment at the Department of Neurosurgery at the “A. Pugliese” Hospital. The presumptive diagnoses were confirmed histologically by the Institute of Pathology after a retrospective review of the three cases. Standard procedures for using the Histolog^®^ scanner for intraoperative diagnosis were followed in all three cases: once the specimen was removed, the fresh tissue was immersed in a fluorescent dye, such as acridine orange, for 10 s and rinsed with saline solution before scanning. The entire procedure, which included examining the specimen on the Histolog^®^ scanner screen, took only a few minutes.

The required equipment was housed and used in the pathology laboratory. Acridine orange staining was performed by the same pathologist who carried out the dissection of the surgical specimen. This specimen had already been used by a laboratory technician to prepare for imprint cytology, which is then stained with hematoxylin and eosin. The pathologist does not require any special observation training because he will see an image on the monitor that is equivalent to a hematoxylin and eosin stain at a magnification of up to approximately 10× under a light microscope. In our series the evaluation of the surgical specimens using Histolog^®^ Scanner was performed collegially by four senior pathologists with a 100% concordance rate. The diagnoses were histologically confirmed by the Institute of Pathology. The three cases were retrospectively reviewed. After intraoperative procedures, materials were treated for paraffin inclusion and routine examination. After intraoperative procedures, materials were treated for paraffin inclusion and routine examination.

## 3. Results

### 3.1. Case 1

**Figure 2:**
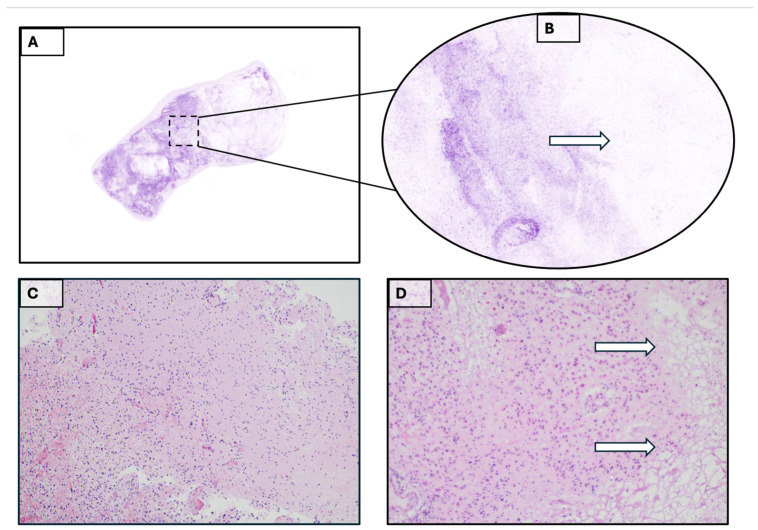
(**A**). Glioblastoma. Basic acquisition of Histolog^®^. (**B**). 100% magnification of the standard acquisition square area. The white arrow indicates the tumor front. (**C**). Hematoxylin-eosin (H&E) stain after embedding. (10×). (**D**). Final Cryostat section with freezing artifacts (arrows), (H&E 10×).

A 69-year-old woman was referred to our hospital due to gait disturbance. Neurological examination (NE) showed a mild left faciobrachial hemiparesis. The patient suffers from severe claustrophobia and is unable to undergo preoperative MRI. Treatment is indicated based on contrast-enhanced cranial CT and whole-body CT (suggesting primary brain tumor). The cranial CT shows an expansive right frontal intraaxial lesion likely extending to the midportion of the corpus callosum [Figure 1A]. The patient underwent surgery through craniotomy for subtotal resection. The intraoperative and definitive histopathological examination allowed us to formulate a diagnosis of glioblastoma wild-type [Figure 2]. Diagnosis was also confirmed by the study of mutational state of IDH1/IDH2 genes.

Specifically, exon 4 of the IDH1 gene (codons 105 and 132) and exon 4 of the IDH2 gene (codons 140 and 172) were examined. The analyses were conducted using the EasyPGX^®^ qPCR Instrument 96 platform and EasyPGX^®^ Analysis Software (Software version 4.0.17, Diatech Pharmacogenetics). The investigation did not reveal the presence of mutations in the exons analyzed. NE at discharge showed an almost complete regression of preoperative hemiparesis.

### 3.2. Case 2

**Figure 3:**
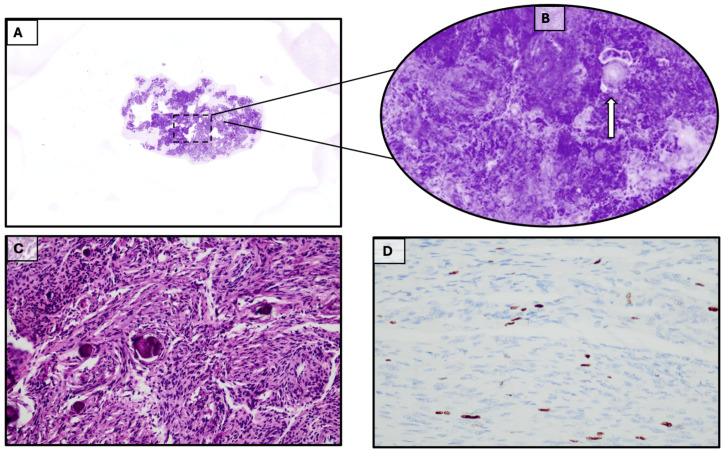
(**A**). Meningioma. Basic acquisition of Histolog^®^. (**B**). 200% magnification: the organoid structure of the neoplasm can be seen. The arrow indicates an onion-bulb psammomatous body. (**C**). H&E staining confirming the accurate reproduction of the neoplasm by Histolog^®^ Scanner (20×). (**D**). Immunohistochemistry confirming the diagnosis of a benign meningiomatous lesion with a low proliferation index (fibrous meningioma).

A 69-year-old woman was admitted to our facility for confusion, behavioral disinhibition, and memory disorder. A brain MRI revealed a meningioma of the left sphenoid wing [Figure 1B]. The patient underwent microsurgical removal of the lesion; after one week, she was discharged in overall improved clinical condition, with persistent mild residual frontal syndrome. The intraoperative and definitive histopathological examination allowed us to formulate a diagnosis of fibrous meningioma [Figure 3].

### 3.3. Case 3

A 71-year-old man, came to our attention following an episode of epileptic seizure, complicated by post-critical aphasia and hemiparesis, which regressed within a few days. Brain MRI showed a left posterior temporal subcortical cystic expansive process located at the junction between the superior temporal gyrus and the angular gyrus [Figure 1C]. 

**Figure 4:**
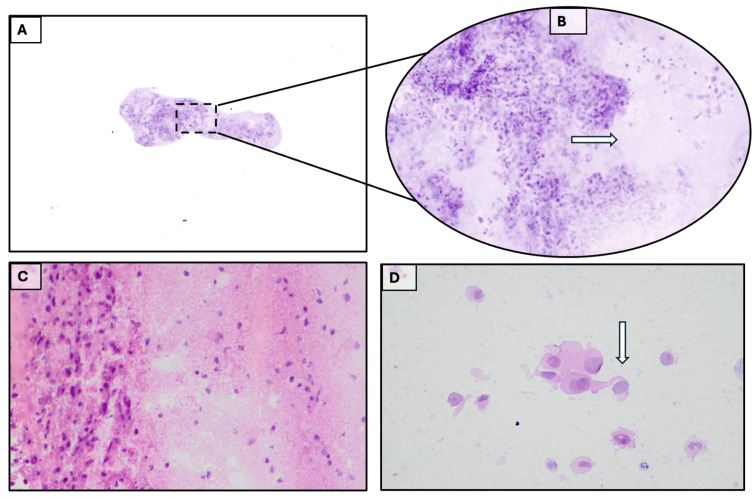
(**A**). Metastasis. Basic acquisition of Histolog^®^. (**B**). 100% magnification of the standard acquisition square area. The arrow indicates the margin between healthy tissue and neoplastic tissue. (**C**). Frozen section showing the border zone between neoplasia and brain tissue, (H&E 10×). (**D**). Extemporaneous cytology by apposition allowing a diagnosis of squamous cell carcinoma to be formulated (arrow on tadpole cells with intercellular bridge), (H&E 40×).

A heterotopic pulmonary finding with lymphadenopathy was also found, suggestive of pulmonary primitivity. On admission, the neurological examination (NE) documented a GCS = 15, a slight nominative aphasia, while the remaining neurological findings were normal. The patient underwent surgery with gross total resection of the lesion. The surgery was performed without perioperative complications, and the patient was discharged on the seventh day. At discharge, the NE revealed a GCS = 15 and moderate mixed aphasia (expressive and sensory). The intraoperative and definitive histopathological examination allowed us to formulate a diagnosis of metastatic squamous cell carcinoma [Figure 4].

## 4. Discussion

The Histolog^®^ Scanner is a device that has gained increasing popularity in recent years, especially in breast and prostate surgery and in the removal of skin lesions. Related scientific articles began to appear on PubMed in 2019 and continue to be cited with increasing frequency up to the present year. The main ones are cited in Table 1. The most important application would seem to be the study of resection margins in oncological surgery, although in some cases it is suggested that the device’s images may be valid for a histopathological diagnosis of the type of lesion. The classic use of the device appears to be in conservative breast surgery (lumpectomy), but further use is also suggested in radical surgery of this organ. In second place in terms of importance is urology, particularly with regard to radical prostatectomy with robotic procedures. Finally, the removal of skin lesions such as basal cell carcinomas, with rapid assessment of the excision margins, would appear to be a third promising field of application. It is therefore possible that this rapid technique, which does not consume tissue material, could be quickly adopted in other surgical fields, including that of the central nervous system.

The evaluation of the three cases studied allows us to hypothesize the use of the Histolog^®^ Scanner in intraoperative neuropathological examination, both with regarding to the issues related to the study of margins and with regarding to the diagnosis of the nature of the lesion. Case 1 highlights that traditional intraoperative histopathological examination can pose problems when establishing a “margin” for gliomas due to the possible presence of freezing artifacts. The Histolog^®^ Scanner can be a resource for this type of need. Interestingly, classifying patients into three groups according the Extension of Resection (EOR) of glioblastoma wild-type as: Supratotal Resection (SupTR), Gross-Total Resection (GTR), and Subtotal Resection (STR) groups, it was evident in Nuclear Magnetic Resonance (NMR) studies that, after surgical treatment, chemoradiotherapy (CRT) and adjuvant Temozolamide chemotherapy, the percentages of local recurrence were SupTR, 57.7%; GTR, 76.0%; STR, 82.8% [19]. We think the use of the Histolog^®^ Scanner, due to its speed and ease of use, can be an important guide to increase the chances of maximizing the extent of surgical resection of malignant brain tumors while avoiding, where possible, collateral damage, obtaining a SupTR. Such an approach could reduce the rate of recurrence after treatment. Case 2 suggests that, although meningiomas often do not require intraoperative diagnosis, examination with this device based on confocal microscopy can provide extremely simple and rapid confirmation of the nature of the lesion. Despite the presumed obviousness of the diagnosis of lesions such as meningiomas from the preoperative phase, sometimes not even the common intraoperative histological and cytological diagnostic techniques allow to can avoid errors consistent with diagnosing lesions of other nature as meningiomas and vice versa [20,21,22,23]. Moreover, also for meningiomas the extent of resection is the principal risk factor for recurrence, and exact knowledge of extent of resection is necessary for prognosis and for planning of adjuvant treatment. Various systems integrate radiological techniques with histopathology and immunohistochemistry [24]. A role for intraoperative use of Histolog^®^ Scanner in such a setting must be assumed. In Case 3, the use of the Histolog^®^ Scanner plus a simple cytological technique such a touch preparation, allows for a complete diagnosis of the lesion and an assessment of its margins without subjecting the tissue to cryostat freezing, with the consequent potential artifacts and loss of material. Margins of resection are quickly and clearly shown through the examination on the screen of the device. Brain metastases occur in 10–30% of cancer patients. Surgical resection is recommended for patients with a good prognosis for patients with a single metastasis [25]. Currently, guidelines recommend additional local therapy with Stereotactic Radiosurgery (SRS) of the resection cavity or combined SRS and whole brain radiation therapy. However, apart from factors such as the EOR and surgical technique used for brain metastasis removal (i.e., en bloc vs. piecemeal resection), the percentage of recurrence in the literature is about 20–40% and this is why the Histolog^®^ Scanner could be an interesting innovation in this field [26]. With regard to the margin issue, in all three cases (i.e., in 100% of the tests), the technique reliably indicated the boundary between neoplastic and healthy tissue. The integration of other techniques, such as imprinting cytology, allows for a definitive diagnosis of the nature of the lesion in a short time and without loss of material, as in the case of metastasis. The response time associated with the use of the Histolog^®^ Scanner alone and its combined use with imprinting cytology is at least a third shorter (a few minutes, less than 5 min) compared to that of cryostat sectioning (about 15 min).

## 5. Conclusions

We believe that the examples provided in this article are not yet sufficient to compare the potential of the Histolog^®^ Scanner with the infinite possibilities of intraoperative neuropathological diagnostics for assessing the nature of the lesion. However, by limiting the comparison to the issue of resection margins for gliomas, metastases, and meningiomas, we have demonstrated that, in this new context, the Histolog Scanner could in the future provide a tool that allows these assessments to be performed without excessively extending the resection to healthy brain tissue or resulting in the loss of diagnostic material.

Regarding a cost-benefit assessment in economic terms, it will only be determined in the future, based on the full range of its applications, which cover all organs and systems, and on how much the use of the instrument will reduce healthcare costs and improve patient clinical outcomes. The data provided in this paper appear promising in this regard.

In conclusion, in our study we suggest the possibility of using the Histolog^®^ Scanner in neuropathology for various purposes including the intraoperative study of tumor margins. However, this paper presents some limitations: the data analyzed provide useful information, but do not allow definitive conclusions to be drawn due to the limited sample size. A more extensive study, including a larger and more diverse sample, could help to strengthen the robustness of the results. The results obtained encourage us to continue on this path.

## Figures and Tables

**Table 1:** Papers cited in PubMed about Histolog^®^ Scanner (HS).

Authors and Date of Publication	Organ Studied	Type of Surgical Specimen	Results	Reference in This Paper
Peters N, Schubert M, Metzler G et al., 2019	Skin	Basal cell carcinoma excision	In the comparison of the HS digital images with the H&E-stained slides, sensitivity was 73%, and specificity was 96%.	[5]
Elfgen C, Papassotiropoulos B, Varga Z et al., 2019	Breast	Fresh breast core needle biopsy	HS provides a visualization of cellular details equivalent to the H&E standards, which could permit rapid diagnosis of malignant and benign breast lesions.	[6]
Grizzetti L, Kuonen F., 2022	Skin	Biopsies and surgical specimens of cutaneous basal cell and squamous cell carcinomas	Ex vivo Confocal Laser Scanning Microscopy (CLSM) is a fast and accurate alternative to analyze surgical margins and ex vivo CLSM using HS is mostly suited for the assessment of non-infiltrative BCCs margins. Especially when conventional histopathological analysis (prone to false negative because of limited margin assessment) is used.	[7]
Sandor MF, Schwalbach B, Hofmann V et al., 2022	Breast	Lumpectomy	Invasive breast cancer and/or in situ-carcinoma lesions can be detected in HS images of surgical margins leading to a potential reduction of 30% and 75% of the re-operations.	[8]
Conversano A, Abbaci M, van Diest P et al., 2023	Breast	Lumpectomy or mastectomy	Ultra-fast confocal microscopy by HS could represent a practical alternative for rapid intraoperative margin assessment of the surface of the whole surgical specimen with conservation of tissue integrity for further histologic and immunologic investigations.	[10]
Togawa R, Hederer J, Ragazzi M et al., 2023	Breast	Lumpectomy	The study demonstrates feasibility of the HS showing similar detection rates for breast cancer compared to the intraoperative standard of care.	[11]
Wernly D, Beniere C, Besse V et al., 2024	Breast	Lumpectomy	Results indicate that the HS is a reliable and time-efficient method for margin assessment	[12]
Abbaci M, Villard A, Auperin A, 2024	Lymph node	Neck lymph node imaging in head and neck surgery	UFCM images by pathologists have a good accuracy (95.5%) for the detection of metastatic lymph nodes in operated patients undergoing sentinel lymph node biopsy or neck dissection with very high specificity (98.8%) but a sensitivity of 76.7% due to the lack of detection of small metastases (<3 mm) and micro metastases (≤2 mm).	[13]
Eissa A, Puliatti S, Rodriguez Peñaranda N, 2024	Prostate biopsies, surgical margins (prostatectomy and cystectomy), kidney biopsies	Review on prostate cancer (15 articles), bladder cancer (1 article), and renal biopsy (1 article).	Ex vivo confocal microscopy (FCM) has been shown to be 85–95% accurate in distinguishing between cancerous and healthy prostate tissue and has been used for intraoperative margin assessment during prostatectomy, reducing the need for frozen sections.	[14]
Cattacin I, Rochat T, Feki A et al., 2025	Breast	Lumpectomy	Training of a surgeon and HS integration into clinical practice are described. When using the training program, 81% and 100% sensitivities were achieved by surgeons.	[9]
Lux MP, Schuller Z, Heimann S, Reichert VMC et al., 2025	Breast	Lumpectomy	The intraoperative use of the HS provides a significant increase in detection rates of lumpectomy positive margins resulting in a substantial reduction in the re-operation rate.	[15]
Almeida-Magana R, Au M, Al-Hammouri T, Mathew M et al., 2025	Prostate	Robot-assisted radical prostatectomy	HS shows a sensitivity of 73–91 and specificity of 94–100% for the presence of Positive Surgical Margins	[16]
Mayor N, Light A, Silvanto A et al., 2025	Prostate	Robot-assisted radical prostatectomy	Study-protocol in progress on margins assessment	[17]
Colard-Thomas J, Pialoux-Guibal A, Gemival P et al., 2025	Breast	Lumpectomy	In Breast Conservative Surgical specimens, the combination of HS and macroscopic examination allowed the accurate assessment of surgical margins and led to intraoperative re-excision in cases where macroscopic examination alone would not have provided an adequate assessment.	[18]

## Data Availability

The original contributions presented in this study are included in the article. Further inquiries can be directed to the corresponding author.

## Abbreviations

The following abbreviations are used in this manuscript:

MRI	Magnetic Resonance Imaging
CT	Computed Tomography
H&E	Hematoxylin-eosin
NE	Neurological Examination
GCS	Glasgow Coma Scale
EOR	Extension Of Resection
SupTR	Supratotal Resection
GTR	Gross-Total Resection
STR	Subtotal Resection
NMR	Nuclear Magnetic Resonance
CRT	Chemoradiotherapy
SRS	Stereotactic RadioSurgery
